# Books Review

**Published:** 1870-07

**Authors:** 


### Books Review.

BARNES'S *Obstetric Operations, with Selections by* BENJAMIN F. DAWSON, M.D. New York: D. APPLETON & Co., 1870.

The profession of this country will gladly welcome the American edition of Dr. Barnes's work on Obstetrics, which is presented nearly in the original form, the editor being aware that it would have been presumptuous to have attempted any improvement of Dr. Barnes's valuable addition to the science of Obstetrics, has therefore simply inserted such notes and additions in the original work as the differences in procedure, and in instruments in this country rendered necessary or advisable.

The work is not intended as a complete work on Obstetrics, being confined to obstetric operations and instruments, the modes of proceedure, and the conditions requiring them. On the subjects treated, it is complete and exceedingly valuable. It is illustrated by numerous wood cuts, showing the relations of parts, and modes of making nearly every Obstetrical operation. This feature of the work commends it as a guide to the practitioner who has had but limited experience in Obstetrical operations; while the sound views of the author, and the valuable additions of the American editor will be sufficient to ensure it a hearty welcome by the American profession. Dr. Barnes enjoys a world wide reputation, and in the department of Obstetrics has no superior.

---

*The Physical Life of Woman: Advice to the Maiden, Wife and Mother, by* George H. Napheys, A.M., M.D. *Published by* George Maclean, *Philadelphia, New York and Boston*; E. Hannaford & Co., *Cincinnati.* 1870.

It has always been a difficult task to write plainly and delicately upon the physical life of either man or woman, and anything like a correct physiological popular work, free from manifest improprieties, has not heretofore been offered the public.

While woman was uneducated it was perhaps possible for her to remain in ignorance of herself, but with general information there must come also some knowledge of her physical life and relations. A sort of general fastidiousness has prevailed in the community which would shut out all knowledge of the sexual functions and relations, but it is very difficult to conceive any objection to the truth, properly expressed, being placed before every woman. There is nothing in the plain scientific facts of our being at all calculated to inflame the passions or corrupt the morals; indeed, quite the reverse. Uneducated imagination or conjecture is a more fruitful source of corruption. Scientific truth, plainly expressed, is safe, and we think the work before us admirably calculated to instruct, and thus purify and ennoble. Dr. Napheys has done a true work for the wives and daughters who care to understand themselves correctly.

---

Chambers, *on Indigestions: on the Diseases of the Digestive Organs functionally treated. Third American Edition.* Henry C. Sea, *Philadelphia,* 1870.

This valuable work on indigestions has long been a standard for the American profession, and it can scarcely be necessary to give any description of it to our readers. The author informs us that since publishing the first edition he has inserted upwards of "ten dozen cases," and re-arranged and in part re-written the comments upon them. This, he says, has greatly augmented the size of the work, which has now probably attained full growth.

Dr. Chambers treats the subjects connected with the indigestions with masterly philosophy and good sense; indeed he carries conviction to his attentive readers. The new edition, though similar to the first and second, is yet more complete, and will be found to contain the most recent and sound views upon all subjects connected with dyspepsia.

---

*Personal Beauty: How to Cultivate and Preserve it, by* D. G. Brinton, M.D., George H. Napheys, M.D., W. J. Holland, *Springfield, Mass.*, 1870.

Personal beauty, a joy for ever. Let us see what there may be in a book on this subject. It tells the power of beauty; what it is, and how to preserve it. It describes the most beautiful figure, and tells how to perfect it, giving the bill of fare how to grow fat or to grow lean. It speaks of the shape of the head, the color of the eye, form and uses of the eyelids, brows and lashes. It describes the proper form of the ear, and the kind of rings to be worn in them; the nose, and the most agreeable smell for it; the mouth and teeth, the breath, and how to sweeten it. It speaks of the arm and hand, and how to beautify them; of the leg and foot, and says that a pretty foot is "the one element of beauty which defies the assaults of age." It gives a chapter on the skin and complexion; the hair and beard; and, speaking of comeliness in general says that "it is the duty of every woman to look as pretty as she possibly can, consistently with her other duties." "*It is a book which should be in the library of every physician.*"

---

*A Physician's Problems, by* Charles Elam, M.D., M.R.C.P. *Boston,* Fields, Osgood & Co., 1869.

This is a book very interesting to read, and very impossible to describe. There is a philosophy about it which is not very easily expressed, but yet runs through it, and impresses and attracts every intelligent reader.

The first chapter is upon "Natural Heritage," in which the author shows how all our essential characters are inherited,—that we bear the traces and consequences of parentage through all our being, in our bodies, souls and spirits. He gives us chapters on "Degenerations in Man;" "Moral and Criminal Epidemics;" "Body and Mind;" "Illusions and Hallucinations; on Somnambulism;" "Revery and Abstraction," &c., &c. The book is worthy of perusal, and is designed for popular reading rather than professional study. It will serve the physician as an entertainment rather than as a practical guide.

Forty

*United States Sanitary Memoirs, by* JOHN A. LIDELL, A.M., M.D. *Edited by* Professor FRANK HASTINGS HAMILTON. *Published for the United States Sanitary Commission, by* HURD & HOUGHTON, 1870.

This volume consists of contributions to the Sanitary Commission, by Dr. Lidell, on "The Wounds of Blood Vessels, *et cetera*; the Traumatic diseases of Bone and Pyæmia." The general subjects of the work are expressed as follows:—1st: On the Wounds of Blood Vessels, Traumatic Hemorrhage, Traumatic Aurism and Traumatic Gangrene. 2nd: On the Secondary Traumatic Lesions of Bone, namely, Osteo-Myelitis, Periostitis, Ostitis, Osteo-Pororis, Cares and Necrosis. 3rd Pyæmia.

These papers contain all the recent observations on the subjects under discussion, together with a large amount of original clinical observation, made by the author while in charge of the Stanton U. S. Army Hospital, where he "began to collect the personal observations, the clinical histories and *post mortem* records, which are set forth in the pages of this work," and some of which are illustrated with beautiful chromo lithographic plates.

The Sanitary commission in the publication of these papers, and the general series, of which this is the first, are conferring a real blessing upon mankind, scarcely less in value and importance than which it so signally accomplished in the first grand work of providing for the sick and disabled soldiers in the time of war.

It is a positive and original contribution to medical science, and will be welcomed by the profession, and appreciated by all who can understand its value.

